# Ultrasound-guided transversalis fascia plane block versus lateral quadratus lumborum plane block for analgesia after inguinal herniotomy in children: a randomized controlled non-inferiority study

**DOI:** 10.1186/s12871-023-02043-x

**Published:** 2023-03-17

**Authors:** Ibrahim Abdelbaser, Doaa Mahmoud Salah, Amer Abdullah Ateyya, Marwa Ibrahim Abdo

**Affiliations:** Department of Anesthesia and Surgical Intensive Care, Faculty of Medicine, Mansoura University, 2 El-Gomhouria Street, Mansoura, 35516 Egypt

**Keywords:** Analgesia, Children, Hernia, Nerve block, Ultrasound, Postoperative

## Abstract

**Background:**

Surgical repair of inguinal hernia is one of the most common day case surgeries in the pediatric population. This study compared the postoperative analgesic effects of transversalis fascia plane block (TFB) versus quadratus lumborum block (QLB) in children scheduled for open unilateral inguinal herniotomy.

**Methods:**

In this prospective, randomized, double-blind, controlled non-inferiority study, 76 eligible patients were recruited. Patients were randomly allocated to either the TFB or QLB group. The primary outcome measure was the proportion of patients who needed rescue analgesia during the first postoperative 12 h. The secondary outcomes were, the time needed to perform the block, the postoperative FLACC score, intraoperative heart rate (HR) and mean arterial pressure (MAP).

**Results:**

The proportion of patients who required a rescue analgesic was comparable (p = 1.000) between the TFB group (7/34, 20.5%) and the QLB group (6/34, 17.6%). The median [Q1-Q3] time needed to perform the block (min) was significantly longer (p < 0.001) in the QLB group (5[5]) compared with the TFB group. The postoperative FLACC pain scale was comparable between the two groups at all-time points of assessment. There is no difference regarding the heart rate and mean arterial blood pressure values at the time points that the values were recorded. (P > 0.005).

**Conclusions:**

Both TFB and QLB similarly provide good postoperative analgesia by reducing the proportion of patients who required rescue analgesia, pain scores and analgesic consumption. Moreover, TFB is technically easier than QLB.

## Background

Surgical repair of inguinal hernia is one of the most common day case surgeries in the pediatric population [1]. Adequate postoperative opioid-free analgesia and early mobilization are basic components of day case surgeries.

Caudal analgesia in lower abdominal surgeries has become unpopular among physicians because of its short duration of analgesia, (4–6) h, and its potential adverse effects including motor block, urine retention and the accidental dural puncture. Currently, fascial blocks are widely used for postoperative analgesia in patients scheduled for lower abdominal surgeries. Transversus abdominis plane block [2], ilioinguinal/iliohypogastric nerve block [3], quadratus lumborum block (QLB) [4], transversalis fascia plane block (TFB) [5], and erector spinae plane block [6] have been used to provide analgesia after pediatric lower abdominal surgeries.

In 2009, Hebbard described ultrasound-guided TFB that targets the T12 and L1 spinal nerves [7]. In TFB the local anesthetic is deposited at the level of posterior axillary line in the layer between the transversus abdominis muscle and its deep investing transversalis fascia [7]. The use of TFB has been reported to be effective in controlling postoperative pain after caesarean delivery [8, 9], inguinal hernia repair [5, 10], harvesting of iliac crest bone graft [11], and pediatric ureteroneocystostomy [12].

The ultrasound-guided QLB was first described by Blanco 2007 in which the local anesthetic is injected in the space between abdominal wall muscles and the lateral margin of the quadratus lumborum muscle [13]. The injected local anesthetic in the potential space for QLB spreads to the thoracic paravertebral space to block the lateral and the anterior cutaneous branches from T7 to L1 [14, 15].

The ultrasound-guided QLB provides effective postoperative analgesia following abdominal surgeries, such as pediatric lower abdominal surgeries [4] caesarean delivery [16], laparoscopic hysterectomy [17], and appendectomy [18].

In this study, we compared the postoperative analgesic effects of TFB versus QLB in children scheduled for open unilateral inguinal herniotomy using the proportion of patients who required rescue analgesia as a primary outcome. We hypothesized that the postoperative analgesia provided by TFB would be non-inferior to QLB.

## Methods

### Ethics

This prospective, randomized, double-blind, controlled non-inferiority study was approved by the Institutional Research Board of the Faculty of Medicine, Mansoura University, Egypt on February 12, 2022, IRB code (MS.22.01.1818). Patients were recruited after registration of the study protocol with the Pan African Clinical Trials Registry (ID: PACTR202203673199781; registration date: 30/03/2022). This study was conducted at Mansura University Children’s Hospital from April to November 2022 in accordance with the Helsinki Declaration 2013 with good clinical practice. Informed written consent was obtained from the parents or the legal guardians of participants the day before surgery.

### Patients

A total of 76 eligible patients were recruited. The inclusion criteria were: (1) patients aged 1–5 years, regardless of sex; (2) patients with American Society of Anesthesiologists physical (ASA) status I or II; (3) patients who underwent elective open surgical repair of unilateral inguinal hernia. The exclusion criteria were: (1) repeated surgeries; (2) history of allergy to bupivacaine and other amide local anesthetics; (3) infection at the site of block needle entry; (4) bleeding diathesis; (5) neurological disorders; (6) ASA ≥ III.

### Randomization and blinding

Patients were randomly allocated to either the TFB or QLB group, in a ratio of 1:1 using SPSS 28 software (Statistical Program for Social Sciences, SPSS Inc., Chicago, Illinois, USA) by a physician who was not involved in the data analysis. Patients group assignments were placed in closed, opaque, sealed and sequentially numbered envelops that were opened in the operating room before induction of anesthesia. Ultrasound-guided TFB or QLB were performed by a single anesthesiologist who have good experience in the ultrasound-guided nerve blocks and was not involved in any other related process according to the patient group allocation. Patients, anesthesiologist who was responsible for patients, surgeon, and physician who was responsible for the postoperative care, all were blinded to patient group allocation.

### Anesthetic management

Patients were premedicated with oral midazolam syrup 0.5 mg/kg an hour before surgery. In the operating room, basic monitoring including pulse oximetry, non-invasive blood pressure cuff and 3 lead electrocardiography were connected to the patient. Anesthesia was induced with 8% sevoflurane in oxygen/air mixture (50%). A 22-gauge venous cannula was inserted to give fentanyl 1 µg/kg, after the patient became deeply anesthetized, an I-Gel supraglottic airway of appropriate size was inserted. Anesthesia was maintained with 1–2% sevoflurane in oxygen/air mixture (50%) and ventilation was assisted spontaneous ventilation. At the end of surgery, the patient received a single dose of intravenous ibuprofen infusion 10 mg/kg.

### Ultrasound-guided TF and QL plane block technique

Ultrasound-guided TFB and QLB were performed in the supine position after securing the airway with I-Gel supraglottic airway by a single anesthesiologist who have good experience in the ultrasound-guided nerve blocks. The skin was sterilized with 10% povidone iodine. The high frequency linear ultrasound transducer of the GE Healthcare Vivid S5 ultrasound machine was also wrapped with a sterilized plastic cover and placed over the skin of the lateral abdominal wall at the midaxillary line midway between the subcostal margin and the iliac crest.

#### A- ultrasound-guided TFB

**Fig. 1:**
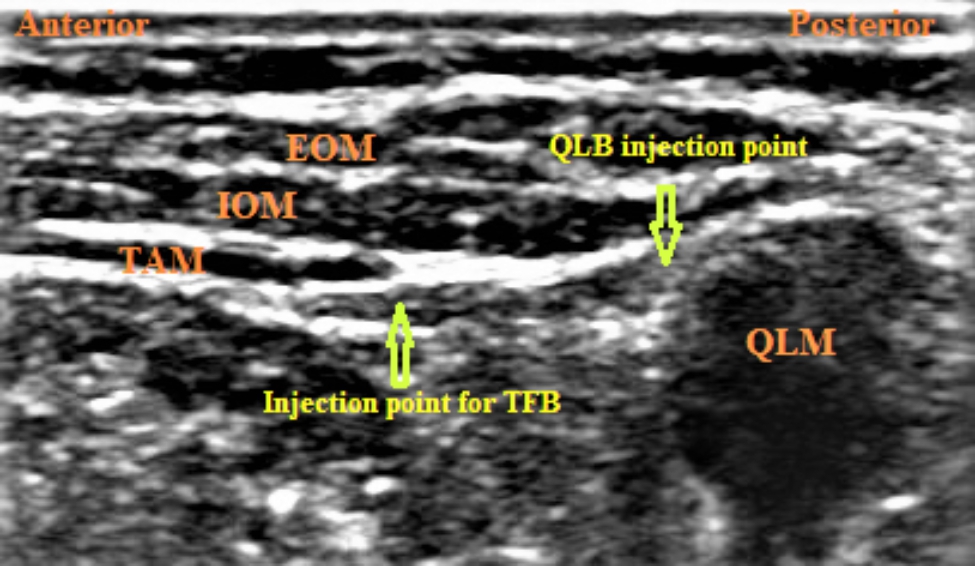
Ultrasonographic images showing the anatomy of abdominal wall muscles and the target points of injection for transversalis fascia plane block (TFB), and quadratus lumborum (QLB); EOM: external oblique muscle; IOM: internal oblique muscle; TAM: transversus abdominis muscle; QLM: quadratus lumborum muscle

The linear transducer mark was directed upward and moved to get an image showing the external oblique muscle, internal oblique muscle, transversus abdominis muscle, the transversalis fascia, and the lateral end of the quadratus lumborum muscle (Fig. 1). A sonographic needle (22G, 50 mm, Stimuplex Ultra, B.Braun Melsungen AG) was introduced through the abdominal wall muscles using in-plane technique to reach the plane between transversus abdominis muscle and transversalis fascia, which was the target point of injection. Then, we injected 0.4 ml/kg bupivacaine 0.25%.

#### B- ultrasound-guided lateral QLB

Lateral QLB was performed following the same steps used in TFB described above, but the target point of needle tip position was the anterolateral border of the quadratus lumborum muscle at its junction with the transversalis fascia, where we injected 0.4 ml/kg bupivacaine 0.25%.

### Postoperative management

The patient stayed in post-anesthesia care unit (PACU) for 30 min, then was transferred to the surgical ward after the full awakening, the control of postoperative pain and thermohemodynamic stability. The FLACC (Face, Leg, Activity, Cry, Consolability) 10 points scale was used for postoperative pain assessment. Intravenous paracetamol 10 mg/kg was given as a rescue analgesic if FLACC pain score was ≥ 4. If FLACC pain score persisted ≥ 4, fentanyl 1 µg/kg was given. All patients were discharged from the hospital after 12 h.

### Outcome measures

The primary outcome measure was the number and proportion of patients who needed rescue analgesia during the first postoperative 12 h. The secondary outcomes were, the time needed to perform the block, the postoperative FLACC score, intraoperative heart rate (HR) and mean arterial pressure (MAP), time to first analgesic request, postoperative paracetamol consumption, the incidence of block-related complications, and parents’ satisfaction score using a 5-point Likert scale [19] (1 = dissatisfied, 2 = slightly satisfied, 3 = moderately satisfied, 4 = very satisfied, 5 = extremely satisfied). The parents’ satisfaction score was assessed at 12 h postoperatively, just before discharge of the patient from hospital. The postoperative FLACC pain score was measured at 30 min in PACU and in surgical ward at 2 h, 4 h, 6 h, 9 h, and 12 h. HR and MAP were recorded at the following time points; basal before induction of anesthesia, after skin incision, during the traction on hernia sac and at the end of surgery. For patients who required rescue analgesia, the time to first analgesic request and paracetamol consumption during the first postoperative 12 h were recorded. The possible complications that might be caused by the block were local hematoma, local anesthetic toxicity, or muscle weakness of lower limbs.

### Sample size estimation and statistical analyses

Sample size was calculated using PASS 15 software. The primary outcome was the proportion of patients who required rescue analgesia in the first postoperative 12 h. We used the results of previous studies in which the proportion of patients who required rescue analgesia in TFP block was 15% (3/20) in TFP block and 12% (3/25) in QLB [4, 5]. We used the Z test for the difference between 2 proportions to calculate the required sample size. Assuming that the non-inferiority margin is 0.25 (25%), the required sample size was 30 patients per group with a power of 80% and clinical significance at 0.05. The number of patients in each was increased to 38 to avoid the probable dropouts.

Data analysis was done using IBM SPSS Statistics for Windows, Version 28.0 (IBM Corp., Armonk, NY, USA). At first, we used Shapiro–Wilk test to evaluate the normality of data distributions. Continuous variables data of normal distribution are expressed as mean ± SD (standard deviation) and variables data of abnormal distribution are expressed as median (interquartile ranges, Q1- Q3). The variables were compared using student’s t-test for data of normal distribution or Mann–Whitney U-for data of abnormal distribution. Categorical variables data are expressed as numbers (percentage) and the Pearson’s chi-square test or Fisher’s exact test was used for their comparison. The statistical significance was considered if P was < 0.05 at a confidence interval of 95%.

## Results

**Fig. 2:**
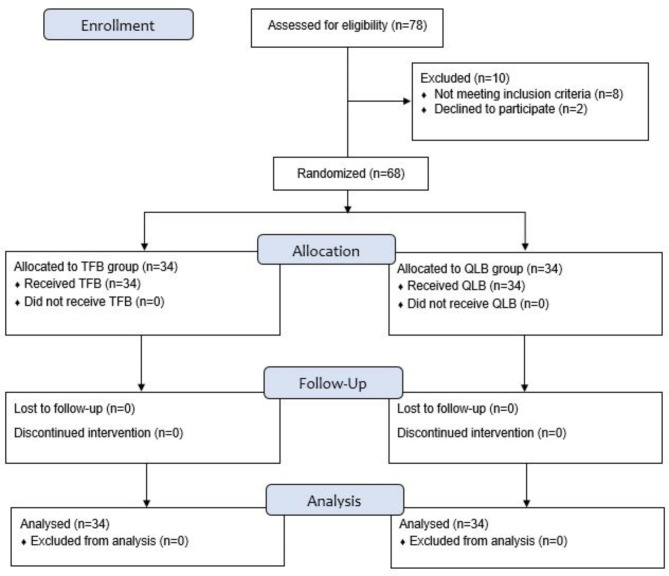
CONSORT Flowchart

We enrolled 78 patients, 10 were excluded and 68 completed the study. The final data analysis was performed on 68 patients, 34 in TFB group and 34 in QLB group (Fig. 2). There were no significant differences in patients’ demographic characteristics (age, gender, height, and weight), duration of surgery and duration of anesthesia between the two groups (Table 1).

**Table 1:** The patient’s demographic data, block time, and duration of anesthesia and surgery

	QLB group (n = 34)	TFB group (n = 34)	P value	95% Confidence Interval
Age, year	3[2–4]	2[2–4]	0.511	
Gender (Male/Female), n	29/5	28/6	1.000	
Weight, kg	15.8 ± 3.9	14.9 ± 3.7	0.330	-0.94, 2.76
Height, cm	93[85–104]	87[84–102]	0.576	
Time needed to perform the block, min	5[5–5]	3[3–3]	< 0.001*	
Duration of surgery, min	44.4 ± 6.1	45.6 ± 5.9	0.404	-4.17, 1.70
Duration of anesthesia, min	58.2 ± 7.3	59.1 ± 6.5	0.516	-4.22, 2.51

Data are expressed as median (Q1-Q3), number (n), mean ± SD (standard deviation)

*P < 0.05 indicates statistical significance

The postoperative analgesic profiles are presented in Table 2. The proportion of patients who required rescue analgesia was comparable (p = 1.000) between the TFB group (7/34, 20.5%) and the QLB group (6/34, 17.6%). The sub-analysis of patients’ data who required rescue analgesia showed that, paracetamol consumption in the first postoperative 12 h and the time to the first analgesic request were comparable between the two groups.

**Table 2:** The number of patients required rescue analgesia, the time of first analgesic request, 12 h postoperative paracetamol consumption for patients who required rescue analgesia and parents’ satisfaction score

	QLB group	TFB group	P value	95% Confidence Interval
Patients needed rescue analgesia n, (%)	6/34 (17.6)	7/34 (20.5)	1.000	(21–28) %
Time to first analgesic request, h	7.1 ± 2.3	7.5 ± 2.3	0.762	-3.27, 2.26
Postoperative paracetamol consumption, mg/kg	13.3 ± 5.1	14.2 ± 5.3	0.751	-7.39, 5.49
Likert parents’ satisfaction scale	4[3–4]	3[3–4]	0.573	

Data are expressed as number (n, %), mean ± SD (standard deviation), median (Q1-Q3).

The median (IQR, [Q1- Q3]) time needed to performs the block (min) was significantly longer (p < 0.001) in the QLB group (5[5]) compared with the TFB group (3[3]) (Table 1). Parents’ satisfaction Likert scale were comparable between the two groups (Table 2). The postoperative FLACC pain scale was comparable between the two groups at all-time points of assessment (Table 3).

**Table 3:** The postoperative FLACC pain score

FLACC measured at:	QLB group (n = 34)	TFB group (n = 34)	P value
30 min in PACU	1[0–2]	1.5[1–2]	0.394
2 h	1[0–2]	1[1–2]	0.497
4 h	1[0–2]	1[1–2]	0.474
6 h	1[1–2]	1[1–2]	0.831
9 h	1[1–2]	1[1–3]	0.467
12 h	2[1–3]	2[1–2]	0.923

Data are expressed as median (Q1- Q3)

There is no difference regarding the heart rate and mean arterial blood pressure values at the time points that the values were recorded. (P > 0.005). (Table 4).

**Table 4:** Intraoperative heart rate (HR) (beat/min) and mean arterial pressure (MAP) (mmhg)

Time point	QLB group (n = 34)	TFB group (n = 34)	P value	95% Confidence Interval
Baseline *HR* *MAP*	117 ± 8 59 ± 6	117 ± 7 59 ± 6	0.766 0.954	-3.33, 4.51 -2.94, 3.11
Skin incision *HR* *MAP*	124 ± 9 62 ± 2	122 ± 7 62 ± 3	0.504 0.950	-2.84, 5.72 -3.84, 3.60
Sac traction *HR* *MAP*	121 ± 11 57 ± 9	119 ± 9 57 ± 2	0.557 0.653	-3.64, 6.70 -2.01, 3.18
End of surgery *HR* *MAP*	118 ± 11 57 ± 6	117 ± 9 56 ± 4	0.695 0.229	-4.19, 6.25 -1.07, 4.43

Data are expressed as mean ± SD (standard deviation)

None of the patients who were included in this study required opioid for postoperative analgesia. The block-related complications (were local hematoma, local anesthetic toxicity or muscle weakness of lower limbs) were not reported in any patient who were included in the study.

## Discussion

This randomized, controlled non-inferiority study compared the postoperative analgesia profile of TFB versus QLB in children undergoing inguinal herniotomy. We did not find statistically significant differences between TFB and QLB in the proportion of patients who required rescue analgesia and the postoperative pain score. However, the time needed to perform TFB was significantly shorter compared with QLB. On sub-analysis of patients’ data who needed rescue analgesia, there were no significant differences in the time to first analgesic request and analgesic consumption between both groups. We did not report the incidence of any complication due to performing TFB or QLB.

In this study, the non-inferiority margin for the proportion of patients who required rescue analgesia was 0.25 (25%) and the 95% confidence interval was (21–28) %. Therefore, the non-inferiority of TFB to QLB was not confirmed as the non-inferiority margin (25%) was lower than the upper 95% confidence interval (28%).

Previous studies have demonstrated that both TFB and QLB are effective in reducing postoperative pain scores and analgesic consumption after inguinal hernia repair [4, 5, 10]. Our previous study has shown the efficacy of TFB in reducing the proportion of patients who required postoperative rescue analgesia, FLACC pain score and paracetamol consumption in children undergoing unilateral inguinal hernia repair [5].

López-González et al. compared the postoperative analgesic efficacy of ultrasound-guided the TFB versus the transversus abdominis plane (TAP) block in adults undergoing outpatient inguinal hernia repair [20]. They found that the proportion of patients who required postoperative rescue analgesia, the total amount of postoperative morphine consumption and pain scores were similar between both groups. However, the TFB was associated with a higher sensory block than TAP block.

An adult study by Fouad et al. used a similar methodology to compare the postoperative analgesia profile of TFB versus QLB in patients scheduled for unilateral inguinal hernia repair [21]. They found that the proportion of patients who required rescue analgesia and the postoperative pain scores were similar between the two groups.

Scimia et al. reported that the ultrasound-guided TFB could provide a satisfactory analgesia and anesthesia of the dermatomes innervated by T12–L1 nerves [22]. The authors suggested that TFB could be used as an alternative to general anesthesia and the standard regional analgesic technique in inguinal hernia repair. Rahimzadeh et al. found that both TFB and TAP block produced similar postoperative analgesic effects and satisfaction rate after elective cesarean Sect. [23].

Öksüz et al. compared the postoperative analgesia of the QLB versus the TAP block in children who underwent open surgery for unilateral inguinal herniotomy or orchiopexy [4]. They found that the proportion of participants who required rescue analgesia in the first postoperative 24 h was significantly lower in the QLB compared with the TAP block. Sørenstua et al. found no difference between QLB and TAP block in postoperative morphine consumption and pain scores after laparoscopic hernia repair in adults [24].

Ragab et al. in a randomized study, compared the analgesic effects and parents’ satisfaction of quadratus block versus the caudal block in children undergoing open inguinal hernia repair [25]. They reported greater analgesic effects and more parents’ satisfaction with QLB than the caudal block. Pang et al. found that QLB combined with oxycodone patient controlled analgesia was associated with good postoperative analgesia and reduced postoperative opioid consumption after laparoscopic hepatectomy [26].

The main sensory innervation of the inguinal region arises from T10-L2 nerves. The ilioinguinal and iliohypogastric nerves originate from T12 and L1 nerves [27]. The hernial sac is innervated by the genital branch of genitofemoral nerve. In TFB, we injected the local anesthetic in the plane between the transversalis fascia and the deep aponeurosis investing the posterior tail of transversus abdominis muscle. Therefore, in TFB, the ilioinguinal and iliohypogastric nerves are blocked early before penetrating the transversus abdominis muscle [28]. There is possibility of the spread of the local anesthetic to the thoracic paravertebral space to block the ventral and dorsal rami of the spinal nerves, therefore, TFB is effective in controlling the visceral pain [29].

The main mechanism of QLB is the spread of the local anesthetic along the thoracolumbar fascia to the paravertebral space to block the spinal nerves [30]. Cadaveric studies [14, 31–33] showed the spread of the injected contrast in cephalad direction to the thoracic paravertebral and intercostal spaces blocking the subcostal, ilioinguinal iliohypogastric nerves and occasionally genitofemoral neve. The caudal spread of the contrast to the lumbar nerves has been reported. The above findings can explain the efficacy of QLB in controlling both somatic and visceral pain.

Consistent with the findings of our study, Fouad et al. [21] found that time needed to perform the TFB was significantly shorter than QLB indicating that the TFB is technically easier than QLB. This could be explained by, in supine position, the target site of local anesthetic injection for TFB is more lateral and anterior than the injection target site for QLB.

The current study demonstrated good control of the hemodynamic response to the intraoperative stressful conditions such as skin incision and traction on the hernia sac in TFB and QLB groups because we performed both blocks before skin incision. Tian et al. used different concentration of ropivacaine for TFB in adult patients undergoing laparotomy and found that the perioperative HR and MAP were more stable and the postoperative pain score was significantly lower with the medium concentration than the low concentration of ropivacaine [34].

In this study, the parents were satisfied from the postoperative analgesia of TFB and QLB because the patients were calm, comfortable and easily sleep. We did not report the incidence of any complications related to the block indicating the safety of both TFB and QLB.

Our study has few important limitations. First, we did not measure the serum concentration of bupivacaine because of the unavailability of this analysis in our hospital. Second, we did not assess the dermatomal sensory spread of TFB and QLB because both blocks were performed under general anesthesia. Third, we did not evaluate the effect of TFB and QLB on the incidence of chronic pain after inguinal hernia repair as this requires the patients follow up for several months. Lastly, we couldn’t accurately comment on the safety of TFB and QLB because of the small sample size, therefore future studies with larger sample size are required.

### Conclusions

Both TFB and QLB similarly provide good postoperative analgesia by reducing the proportion of patients who required rescue analgesia, pain scores and analgesic consumption. Moreover, TFB is technically easier than QLB.

## Data Availability

The datasets used and/or analyzed during the current study are available from the corresponding author on reasonable request.

## Declarations

### Clinical trial registry

Pan African Clinical Trials Registry (ID: PACTR202203673199781; registration date: 30/03/2022).

## List to abbreviations

QLB: Quadratus lumborum block
TFB: Transversalis fascia block
ASA: American Society of Anesthesiologists
PACU: post-anesthesia care unit
FLACC: Face, Leg, Activity, Cry, Consolability
TAP: Transversus abdominis plane
EOM: External oblique muscle
IOM: Internal oblique muscle

## References

[1] Baird R, Guilbault MP, Tessier R, Ansermino JM (2013). A systematic review and meta-analysis of caudal blockade versus alternative analgesic strategies for pediatric inguinal hernia repair. J Pediatr Surg.

[2] Bryskin RB, Londergan B, Wheatley R, Heng R, Lewis M, Barraza M (2015). Transversus Abdominis Plane Block Versus Caudal Epidural for Lower Abdominal surgery in children: a double-blinded Randomized Controlled Trial. Anesth Analg.

[3] Abdellatif AA (2012). Ultrasound-guided ilioinguinal/iliohypogastric nerve blocks versus caudal block for postoperative analgesia in children undergoing unilateral groin surgery. Saudi J Anaesth.

[4] Öksüz G, Bilal B, Gürkan Y, Urfalioğlu A, Arslan M, Gişi G (2017). Quadratus Lumborum Block Versus Transversus Abdominis Plane Block in Children undergoing low abdominal surgery: a Randomized Controlled Trial. Reg Anesth Pain Med.

[5] Abdelbaser I, Mageed NA, El-Emam EM, ALseoudy MM, Elmorsy MM (2021). Preemptive analgesic efficacy of ultrasound-guided transversalis fascia plane block in children undergoing inguinal herniorrhaphy: a randomized, double-blind, controlled study. Korean J Anesthesiol.

[6] Aksu C, Şen MC, Akay MA, Baydemir C, Gürkan Y (2019). Erector Spinae Plane Block vs Quadratus Lumborum Block for pediatric lower abdominal surgery: a double blinded, prospective, and randomized trial. J Clin Anesth.

[7] Hebbard PD (2009). Transversalis fascia plane block, a novel ultrasound-guided abdominal wall nerve block. Can J Anaesth.

[8] Serifsoya TE, Tulgara S, Selvia O, Senturka O, Ilterb E, Pekerb BH (2020). Evaluation of ultrasound-guided transversalis fascia plane block for postoperative analgesia in cesarean section: a prospective, randomized,controlled clinical trial. J Clin Anesth.

[9] Aydin ME, Bedir Z, Yayik AM, Celik EC, Ates İ, Ahiskalioglu EO (2020). Subarachnoid block and ultrasound-guided transversalis fascia plane block for caesarean section: a randomised, double-blind, placebo-controlled trial. Eur J Anaesthesiol.

[10] Ahiskalioglu A, Aydin ME, Doymus O, Yayikb AM, Celik EC (2019). Ultrasound guided transversalis fascia plane block for lower abdominal surgery: first pediatric report. J Clin Anesth.

[11] Black ND, Malhas L, Jin R, Bhatia A, Chan VWS, Chin KJ (2019). The analgesic efficacy of the transversalis fascia plane block in iliac crest bone graft harvesting: a randomized controlled trial. Korean J Anesthesiol.

[12] Peksöz U, Yayık AM, Çelik EC (2022). Efficacy of ultrasound-guided transversalis fascia plane block in pediatric ureteroneocystostomy surgery. Korean J Anesthesiol.

[13] Blanco R (2007). TAP block under ultrasound guidance: the description of a ‘non pops technique. Reg Anesth Pain Med.

[14] Carline L, McLeod GA, Lamb C (2016). A cadaver study comparing spread of dye and nerve involvement after three different quadratus lumborum blocks. Br J Anaesth.

[15] Yang HM, Park SJ, Yoon KB, Park K, Kim SH. Cadaveric evaluation of different approaches for quadratus lumborum blocks. Pain Res Manag 2018; 2018: 2368930. 10.1155/2018/2368930 PMC6016158 29991972

[16] Hansen CK, Dam M, Steingrimsdottir GE, Laier GH, Lebech M, Poulsen TD, et al. Ultrasound-guided transmuscular quadratus lumborum block for elective cesarean section significantly reduces postoperative opioid consumption and prolongs time to first opioid request: a double-blind randomized trial. Reg Anesth Pain Med. 2019 Jul;14:rapm–2019. 10.1136/rapm-2019-100540 31308263

[17] Singh N, Rao PB, Elayat A (2021). Ultrasound-guided anterior and posterior quadratus lumborum block for analgesia after laparoscopic hysterectomy. Pain Manag.

[18] Mullins CF, O’Brien C, O’Connor TC. Novel use of combination of electromyography and ultrasound to guide quadratus lumborum block after open appendicectomy. BMJ Case Rep. 2017 May15;2017:bcr2017219680. doi: 10.1136/bcr-2017-219680. 10.1136/bcr-2017-219680 PMC5753701 28512101

[19] Gill M, Drendel AL, Weisman SJ (2013). Parent satisfaction with acute pediatric pain treatment at home. Clin J Pain.

[20] López-González JM, López-Álvarez S, Jiménez Gómez BM, Areán González I, Illodo Miramontes G, Padín Barreiro L (2016). Ultrasound-guided transversalis fascia plane block versus anterior transversus abdominis plane block in outpatient inguinal hernia repair. Rev Esp Anestesiol Reanim.

[21] Fouad AZ, Abdel-Aal IRM, Gadelrab MRMA, Mohammed HMES (2021). Ultrasound-guided transversalis fascia plane block versus transmuscular quadratus lumborum block for post-operative analgesia in inguinal hernia repair. Korean J Pain.

[22] Scimia P, Basso Ricci E, Petrucci E, Behr AU, Marinangeli F, Fusco P (2018). Ultrasound-guided transversalis fascia plane block: an alternative approach for anesthesia in inguinal herniorrhaphy: a case report. A A Pract.

[23] Rahimzadeh P, Faiz SHR, Imani F, Rahimian Jahromi M (2018). Comparison between ultrasound guided transversalis fascia plane and transversus abdominis plane block on postoperative pain in patients undergoing elective cesarean section: a randomized clinical trial. Iran Red Crescent Med J.

[24] Sørenstua M, Raeder J, Vamnes JS, Leonardsen AL. Efficacy of a TAP block versus an anterior QLB for laparoscopic inguinal hernia repair: a randomised controlled trial. Acta Anaesthesiol Scand. 2022 Oct;20. 10.1111/aas.14160. 10.1111/aas.14160 PMC10092777 36267030

[25] Ragab SG, El Gohary MM, Abd El baky DL, Nawwar KMA (2022). Ultrasound-guided Quadratus Lumborum Block Versus Caudal Block for Pain Relief in Children Undergoing Lower Abdominal Surgeries: a Randomized, double-blind comparative study. Anesth Pain Med.

[26] Pang M, Sun G, Yao W, Zhou S, Shen N, Liao H, Xie H, Gao W, Ge M. Ultrasound-guided transmuscular quadratus lumborum block reduced postoperative opioids consumptions in patients after laparoscopic hepatectomy: a three-arm randomized controlled trial. BMC Anesthesiol. 2021 Feb 11;21(1):45. doi: 10.1186/s12871-021-01255-3. 10.1186/s12871-021-01255-3 PMC7877010 33573598

[27] Fredrickson MJ, Paine C, Hamill J (2010). Improved analgesia with the ilioinguinal block compared to the transversus abdominis plane block after pediatric inguinal surgery: a prospective randomized trial. Paediatr Anaesth.

[28] Konschake M, Zwierzina M, Moriggl B, Függer R, Mayer F, Brunner W (2020). The inguinal region revisited: the surgical point of view: an anatomical-surgical mapping and sonographic approach regarding postoperative chronic groin pain following open hernia repair. Hernia.

[29] Carney J, Finnerty O, Rauf J, Bergin D, Laffey JG, Mc Donnell JG (2011). Studies on the spread of local anaesthetic solution in transversus abdominis plane blocks. Anaesthesia.

[30] Murouchi T, Iwasaki S, Yamakage M (2016). Quadratus lumborum block: analgesic effects and chronological ropivacaine concentrations after laparoscopic surgery. Reg Anesth Pain Med.

[31] Dam M, Moriggl B, Hansen CK, Hoermann R, Bendtsen TF, Børglum J. The pathway of injectate spread with the transmuscular quadratus lumborum block: a cadaver study.Anesth Analg; 125 (1):303–12. 10.1213/ANE.0000000000001922 28277325

[32] Adhikary SD, El-Boghdadly K, Nasralah Z, Sarwani N, Nixon AM, Chin KJ (2017). A radiologic and anatomic assessment of injectate spread following transmuscularquadratus lumborum block in cadavers. Anaesthesia.

[33] Elsharkawy H, El-Boghdadly K, Kolli S, Esa WAS, DeGrande S, Soliman LM (2017). Injectate spread following anterior subcostal and posterior approaches to the quadratus lumborum block: a comparative cadaveric study. Eur J Anaesthesiol.

[34] Tian Y, Zhan Y, Liu K, Bu S, Tian Y, Xiong C, Shen J. Analgesic effects of different concentrations of ropivacaine in transversalis fascia plane block during laparotomy. BMC Anesthesiol. 2022 Feb26;22(1):54. doi: 10.1186/s12871-022-01595-8. 10.1186/s12871-022-01595-8 PMC8881832 35219302

